# Pluronic F127-Modified Electrospun Fibrous Meshes for Synergistic Combination Chemotherapy of Colon Cancer

**DOI:** 10.3389/fbioe.2020.618516

**Published:** 2021-02-16

**Authors:** Dengchao Xie, Panpan Ma, Xin Ding, Xiao Yang, Lian Duan, Bo Xiao, Shixiong Yi

**Affiliations:** ^1^State Key Laboratory of Silkworm Genome Biology, College of Sericulture, Textile and Biomass Sciences, Southwest University, Chongqing, China; ^2^College of Food Science, Southwest University, Chongqing, China; ^3^Chemical and Biological Technologies for Health Unit, School of Pharmacy, CNRS UMR8258, INSERM U1267, Université de Paris, Paris, France; ^4^Ministry of Agriculture and Rural Affairs Key Laboratory of Sericultural Biology and Genetic Breeding, College of Sericulture, Textile and Biomass Sciences, Southwest University, Chongqing, China

**Keywords:** pluronic F127, electrospining, fibrous mesh, colon cancer, combination chemotherapy

## Abstract

Colon cancer ranks as the third most common malignancy in the world. Combination chemotherapy, resorting to electrospun fibrous technology, has been considered as a promising strategy to exert synergistic effects in colon cancer treatment. Herein, we manufactured various pluronic F127 (PF127)-modified electrospun fibrous meshes with different weight ratios of camptothecin (CPT) and curcumin (CUR). The fluorescence characterization of the obtained PF127-CPT-meshes, PF127-CUR-meshes, and PF127-CPT/CUR-meshes (2:1) showed that CPT and CUR were evenly distributed within individual fibers of these meshes. Drug release experiments revealed that both types of drugs could be released from fibrous meshes simultaneously and sustainably. Importantly, these meshes exhibited strong *in vitro* anti-colon cancer activities, compared with the control meshes without drugs. Moreover, the combination index values of the PF127-CPT/CUR-meshes (CPT/CUR weight ratio = 5:1, 3:1, or 2:1) were <0.5 after incubation for respective 24 and 36 h, indicating the synergistic anti-colon cancer effects of CPT and CUR in fibrous meshes. Collectively, these results demonstrate that PF127-CPT/CUR-meshes can be developed as an efficient implantable system for effective synergistic treatment of colon cancer.

## Introduction

Colon cancer is one of the most common malignancies around the world, which is associated with high morbidity and mortality (The Colon Cancer Laparoscopic or Open Resection Study Group, 2009; Arnold et al., 2017). In most cases, surgery is performed to remove colon tumor tissues, and chemotherapy has been regarded as an critical supplement with surgery aiming at reducing the harm of cancer recurrence (Arredondo et al., 2017; Ma et al., 2018). Unfortunately, the strategies often induce non-specific systematic distribution of chemic drugs, resulting in rapid blood clearance, drug resistance, and systemic adverse effects (Lashof-Sullivan et al., 2019). Furthermore, a single chemotherapeutic agent falls short of ideal therapeutic effect due to tumor heterogeneity, drug resistance, and low therapeutic effect (Dylla et al., 2008).

Recently, combination chemotherapy has been proposed as an effective approach for cancer therapy, as it offers numerous advantegous features such as enhancement of genetic barriers for tumor cell mutations, synergistic anti-cancer efficacy, and decrease of total drug amounts (Ha et al., 2013; Ramasamy et al., 2014). Camptothecin (CPT), a hydrophobic natural alkaloid, is found in *Camptotheca acuminate* with strong anti-cancer activity (Martino et al., 2017). In the perspective of anti-cancer mechanism, it turns topoisomerase I into a cellular poison by restraining the movement of the replication fork, leading to the death of tumor cells (Darpa et al., 1990). Despite CPT exerts impressive preclinical anti-cancer effect, its further clinical translation has been limited by low treatment efficacy and serious adverse effects (Liu et al., 2009). Curcumin (CUR), a typical natural yellow compound extracted from turmeric, is well-known for its diverse pharmacological effects (e.g., anti-oxidantion, anti-inflammation, and anti-cancer). It inhibited the growth of tumor cells through the nuclear factor kappa B and Wnt signaling pathways (Baliga et al., 2012). However, its clinical application has been seriously constrained by several drawbacks, including poor solubility, low bioavailability, and high metabolism rate (Nelson et al., 2017).

Nowadays, drug delivery systems (DDSs) such as liposomes, hydrogels, and fibrous meshes have been exploited as feasible platforms for controlled drug release, maximized therapeutic efficacy, and minimized side effects (Wiranowska et al., 2019). Among these DDSs, electrospun fibrous meshes appear as one of the most versatile and scalable DDSs for localized chemotherapy. The electrospinning technique has the advantages of being mild and relatively straightforward (Mirjalili and Zohoori, 2016; Ma et al., 2019). Moreover, characterized by their large specific surface area, electrospun fibrous mesh has been considered as a multi-faceted and malleable DDS, which has the superiority of transformation to suitable shapes and specific sizes catering to the post-resection space of tumor sites, balance of the weight ratios of the loaded drugs, and reservation of their anti-cancer activities (Xie et al., 2010).

Poly(lactic acid/glycolic acid) (PLGA), an FDA-approved polymer, has attracted increasing interest as a drug delivery material due to its excellent biocompatibility, desirable biodegradability, and flexible mechanical properties (Hu et al., 2013; Han et al., 2017). Pluronic copolymer is another FDA-approved amphiphilic material (Xiao et al., 2015a). Our recent report demonstrated that pluronic F127 (PF127) could improve the surface hydrophilicity, reduce protein adsorption, modulate drug release rate of fibrous meshes, and further enhance their localized chemotherapeutic effects (Ma et al., 2019). However, to the best of our knowledge, no attempt has been conducted to study the effects of combination chemotherapy against colon cancer on the basis of PLGA/PF127-based fibrous meshes. In this investigation, PF127-modified CPT/CUR-loaded PLGA-based fibrous meshes (PF127-CPT/CUR-meshes) were prepared by electrospinning. We further characterized their physicochemical properties and drug release profiles. In addition, their *in vitro* synergistic anti-cancer activities were evaluated.

## Materials and Methods

### Materials

PLGA (lactide:glycolide = 1:1, Mw = 44 kDa) was obtained from Jinan Daigang Biomaterial Co., Ltd. (Jinan, China). PF127, CPT, CUR, dichloromethane (DCM), and dimethyl sulfoxide (DMSO) were supplied by Sigma-Aldrich (St. Louis, USA). Methanol, 3-(4,5-dimethylthiazol-2-yl)-2,5-diphenyltetrazolium bromide (MTT), and Triton X-100 were supplied by Aladdin (Shanghai, China). All commercial products were used without further purification.

### Fabrication of Electrospun Fibrous Meshes

Fibrous meshes were fabricated by electrospinning technique. The detailed experimental procedure followed our previous report (Ma et al., 2019). PLGA (0.8 g), PF127 (0.2 g), and CPT/CUR were co-dissolved in 6 mL of DCM/methanol mixture systems (8:2, v/v) to obtain the mixed electrospinning solution. A high electric potential was used to a droplet of electrospinning solution at the tip (ID 0.51 mm) of a flat-end needle (Beijing Machinery and Electricity Institute, Beijing, China). During the process, a high voltage of 20 kV and a controllable feed rate of 1.0 mL/min were applied. The electrospinning process was carried out at 25°C and 50% humidity. Finally, the fibrous meshes were collected, vacuum dried, and stored at −4°C.

### Physicochemical Characterizations of Meshes

The viscosity of the electrospun solution was measured at 25°C by using Anton pear Dynamic Shear Rheomete (Austria). The conductivity of the solutions was measured at 25°C by using DDS-11A conductometer (Shanghai Linda instrument, China). Mesh morphology was observed using a scanning electron microscope (SEM, JSM-6510LV, Japan). Afterward, the average fiber diameters were calculated by the analysis of the images with Image J software. Fifty fibers per image were chosen at random to estimate the average and standard deviation of fiber diameters. The distribution profiles of CPT and CUR in meshes were imaged by a confocal laser scanning microscope (Zeiss LSM 800, Germany). The X-ray diffraction (XRD) spectra of various meshes were measured (XRD-7000, Shimadzu, Japan) by scanning from 10 to 50° at a speed of 5 degrees per min and operating at 40 kV and 30 mA.

Thermal gravimetric analysis (TG) of meshes was determined on Q500 TGA instrument (TA Instruments, New Castle, USA). Meshes were heated to 600°C from 20°C at 10°C/min in nitrogen atmosphere. The loading amount and encapsulation efficiency of CPT and CUR in meshes were determined according to our previous report (Xiao et al., 2015b). Briefly, meshes were dissolved in DMSO, and the obtained solution was analyzed using a fluorescence spectrophotometer (RF-5301 PC, Shimadzu, Japan). The emission fluorescence intensity of CPT was measured at 430 nm with an excitation wavelength of 360 nm while that of CUR was measured at 530 nm under an excitation wavelength of 425. The equations of drug loading and encapsulation efficiency were obtained as follows:

$$\begin{array}{l} \text{Drug }\text{loading}=\frac{\text{Weight of actual drugs in fibrous meshes}}{\text{Weight of fibrous meshes}}\times 100\% \end{array}$$

$$\begin{array}{l} \text{Encapsulation efficiency}=\frac{\text{Weight of actual drugs in fibrous meshes}}{\text{Weight of fed drug}}\times 100\% \end{array}$$

### Wettability of Meshes

Water contact angles were obtained by a contact angle goniometer (KRUSS, DSA II GmbH, Germany) at ambient temperature. Meshes were cut into square strips with a size of 1 × 1 cm^2^ for measurement of contact angles. The droplet images were obtained by high-speed camera, and the contact angles were also determined.

### Degradation of Meshes

The degradation profiles of meshes were studied following a previous report (Ma et al., 2019). Briefly, various meshes were placed in simulated colonic fluid (pH 6.2), and the mesh-contained centriguge tubes were deposited in a water bath shaking at 120 rpm at 37°C. After incubation for 24 and 49 days, the meshes were rinsed with water and further dried in a fume hood overnight. Their morphologies were detected using a SEM.

### Drug Release Profiles of Meshes

The release profiles of CPT and CUR from PF127-CPT/CUR-meshes (2:1) were determined using a dialysis method. Eight mg of fibrous mesh was dispersed in 2 mL of simulated colonic fluid (pH 6.2). Thereafter, the suspension was added into dialysis bags (MWCO = 8–14 kDa), which were immersed in the 20 mL of simulated colonic fluid (pH 6.2) at 150 rpm. Tween-80 was added into the releasing media to maintain the solubility of CPT and CUR. At a given time, the outer solution was withdrawn, and the fresh releasing media were added. The amounts of drugs in the releasing media were quantified using a microplate reader (Biotek Instruments, USA).

### Anti-colon Cancer Activities of Meshes

MTT assays were perfomed to investigate the anti-colon cancer activities of meshes. After exposure to meshes at different time periods, CT-26 cells were incubated with MTT solution (0.5 mg/mL, 100 μL) at 37°C for 4 h. Subsequently, the supernatant was removed, and DMSO (80 μL) was added for spectrophotometric measurements at 570 nm using a Tecan SPARK-10 M Plate Reader (Crailsheim, Germany). The blank PF127-mesh was used as a negative control, whereas cells treated with Triton X-100 solution (0.5%, w/v) were applied as a positive control. Finally, cell viabilities were used to determine the combination index (CI) values, which revealed the interaction of two drugs using CalcuSyn software (Biosoft, Cambridge, UK). CI values ranging from 0.9 to 1.1 indicated additive activity. CI values <0.9 and CI values >1.1 represented synergy effect and drug antagonism, respectively.

### Statistical Analysis

Statistical analysis was carried out using ANOVA test followed by Student's *t*-test. Results are expressed as the mean ± standard error of the mean (S.E.M.). Statistical significance was expressed by ^*^p < 0.05 and ^**^p < 0.01.

## Results and Discussion

### Physicochemical Characterization of Meshes

**Figure 1:**
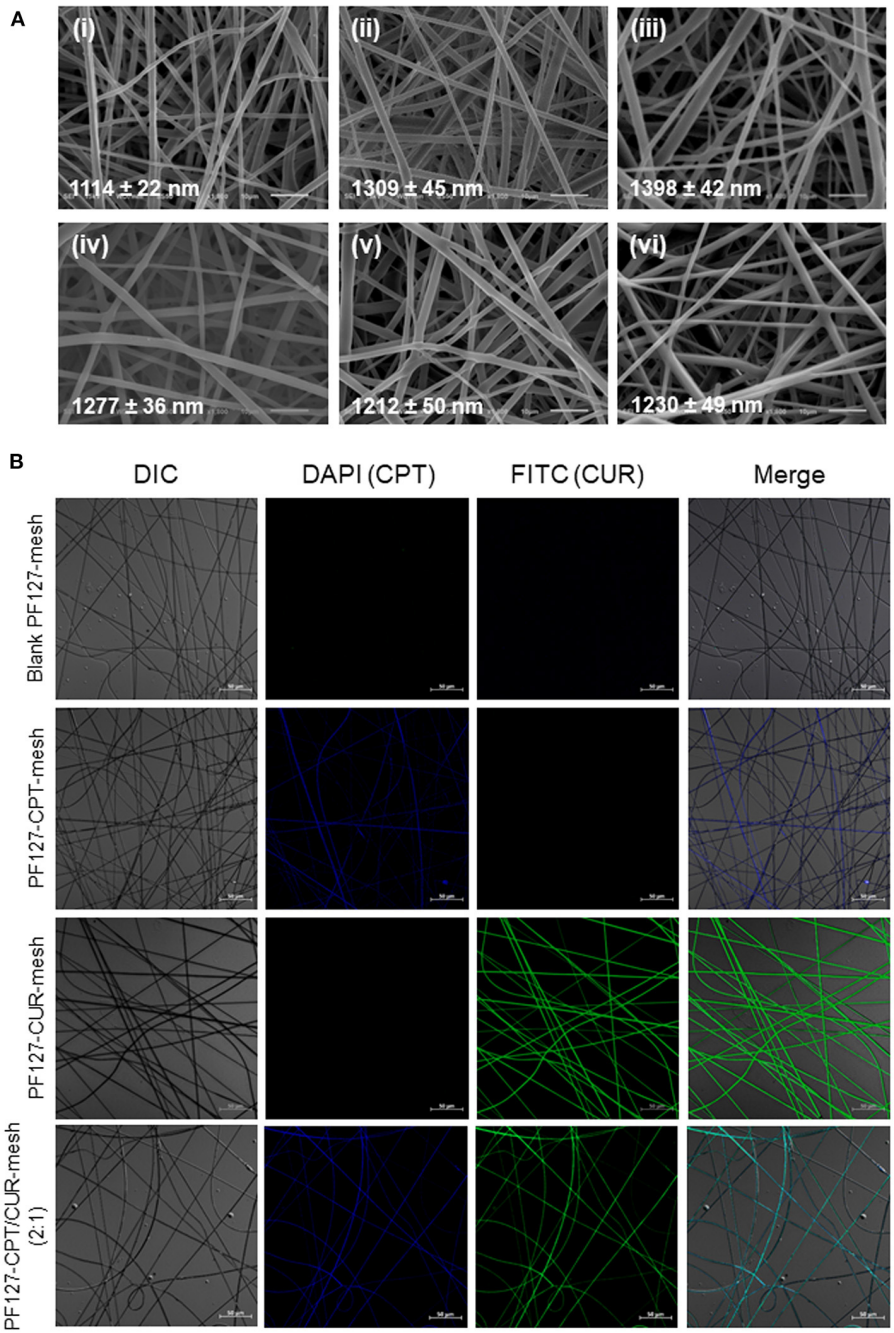
**(A)** SEM images of (i) blank PF127-mesh, (ii) PF127-CPT-mesh, (iii) PF127-CUR-mesh, (iv) PF127-CPT/CUR-mesh (5:1), (v) PF127-CPT/CUR-mesh (3:1), and (vi) PF127-CPT/CUR-mesh (2:1). **(B)** Fluorescence images of various meshes.

Since viscosity and conductivity have important effects on the morphology of electrospun fibers (Thompson et al., 2007), we determined these two parameters. We found that the viscosity and conductivity of electrospun solution were 247.9 mPa·s (shear rate: 25.3 s^−1^) and 4.9 μS/cm. As seen in Figure 1A, fibers in all the electrospun fibrous meshes exhibited integrated properties of an uniform, bead-free, and smooth surface, and the average diameters of these fibers were ranging from 1,114 to 1,398 nm. It was worth noting that no obvious drug crystal was observed on the surface of fibers, indicating that the hydrophobic drugs (CPT and CUR) might be encapsulated in the interior of fibers. To verify this point, the fluorescence images of various meshes were obtained by using a confocal laser scanning microscope (Figure 1B). It was obvious that the blank PF127-mesh exhibited no fluorescence signals. On the contrary, the fibers in the PF127-CPT-mesh and PF127-CUR-mesh emitted blue fluorescent light and green fluorescent light, respectively. In terms of PF127-CPT/CUR-mesh (2:1), both blue and green fluorescence signals were detected from its fibers. These observations demonstrate the drugs (CPT and CUR) are well-distributed within the fibers during the electrospinning process. As summarized in Table 1, the drug loading values of these fibrous meshes ranged from 1.4 to 6.7%, and CPT had a much higher drug loading value than CUR. In addition, we found that the encapsulation efficiency values mainly depended on the types and numbers of drugs dissolved in the electrospinning solution. For instance, the single drug-contained meshes had obviously higher encapsulation efficiency values than the dual drug-contained meshes, which was in agreement with our previous reports (Xiao et al., 2015c).

**Table 1:** Parameters of different fibrous meshes.

**Fibrous mesh**	**Drug loading (%)**		**Encapsulation efficiency (%)**	
	**CPT**	**CUR**	**CPT**	**CUR**
PF127-CPT-mesh	6.7 ± 0.1	–	90.7 ± 2.1	–
PF127-CUR-mesh	–	3.8 ± 0.1	–	50.9 ± 3.1
PF127-CPT/CUR-mesh (2:1)	3.0 ± 0.1	1.4 ± 0.1	48.7 ± 1.1	40.8 ± 1.5

### Wettability and Degradation of Meshes

**Figure 2:**
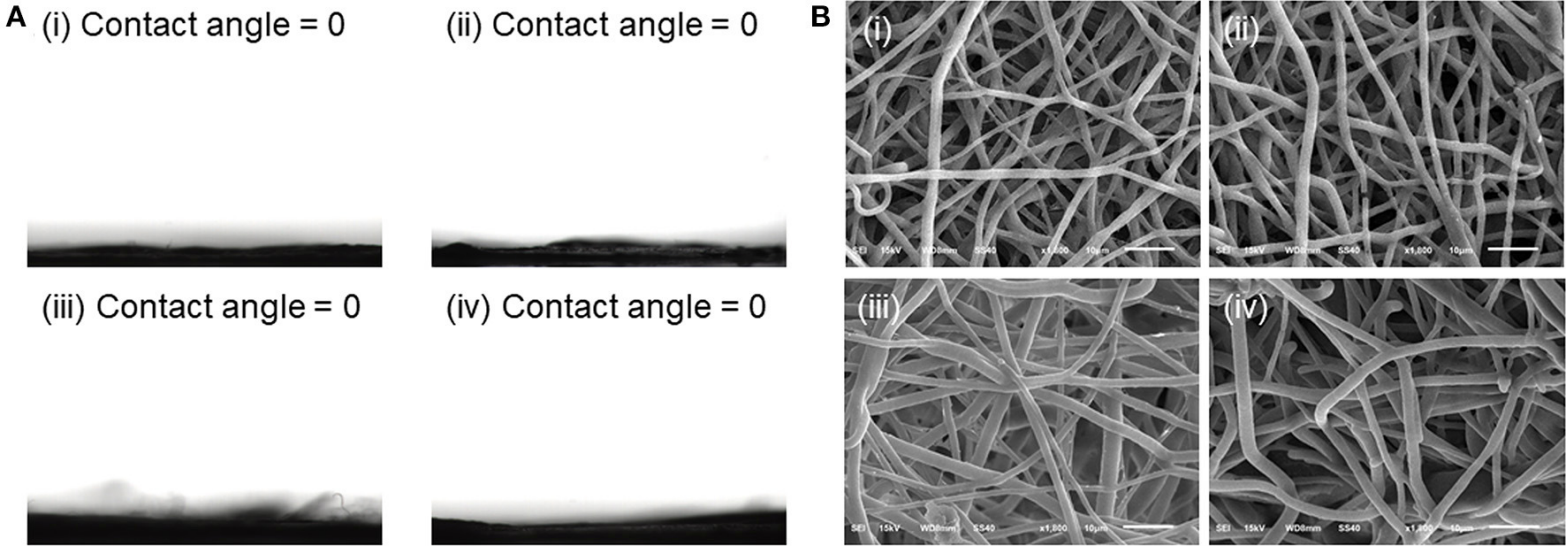
**(A)** Water contact angles of (i) blank PF127-mesh, (ii) PF127-CPT-mesh, (iii) PF127-CUR-mesh, and (iv) PF127-CPT/CUR-mesh (2:1). **(B)** SEM images of various meshes after incubation in simulated colonic fluid (pH 6.2) for 24 days: (i) blank PF127-mesh, (ii) PF127-CPT-mesh, (iii) PF127-CUR-mesh, and (iv) PF127-CPT/CUR-mesh (2:1).

Wettability was reported to have an important impact on the protein absorption of fibrous meshes (Kim et al., 2003). Therefore, water contact angle was determined to study the wettability of different meshes. Our previous study demonstrated that PLGA mesh was hydrophobic and had a contact angle of around 117.6° (Ma et al., 2019). However, with the introduction of PF127 to the electrospinning solutions, the water droplets were absorbed within several seconds, and all the fibrous meshes showed the water contact angles of 0°, which could be attributed to the presence of hydrophilic PEO of PF127 on the surface of fibrous meshes (Figure 2A).

Since PF127-modified meshes had a highly hydrophilic surface, the degradation profiles of these fibrous meshes were measured in simulated colonic fluid (pH 6.2). Figure 1A showed that the individual fibers of all the meshes were relatively straight. However, after incubation for 24 days, these fibers became tortuous and swollen, and the interstitial space among these fibers was greatly reduced (Figure 2B). Furthermore, we observed that after incubation in simulated colonic fluid (pH 6.2) for 49 days, the morphology of PF127-CPT/CUR-mesh (2:1) transformed from individual fibers to disintergrated states (Supplementary Figure 1), indicating that the meshes were degraded after 49 days of incubation.

### XRD Patterns and Drug Release Profiles of Meshes

**Figure 3:**
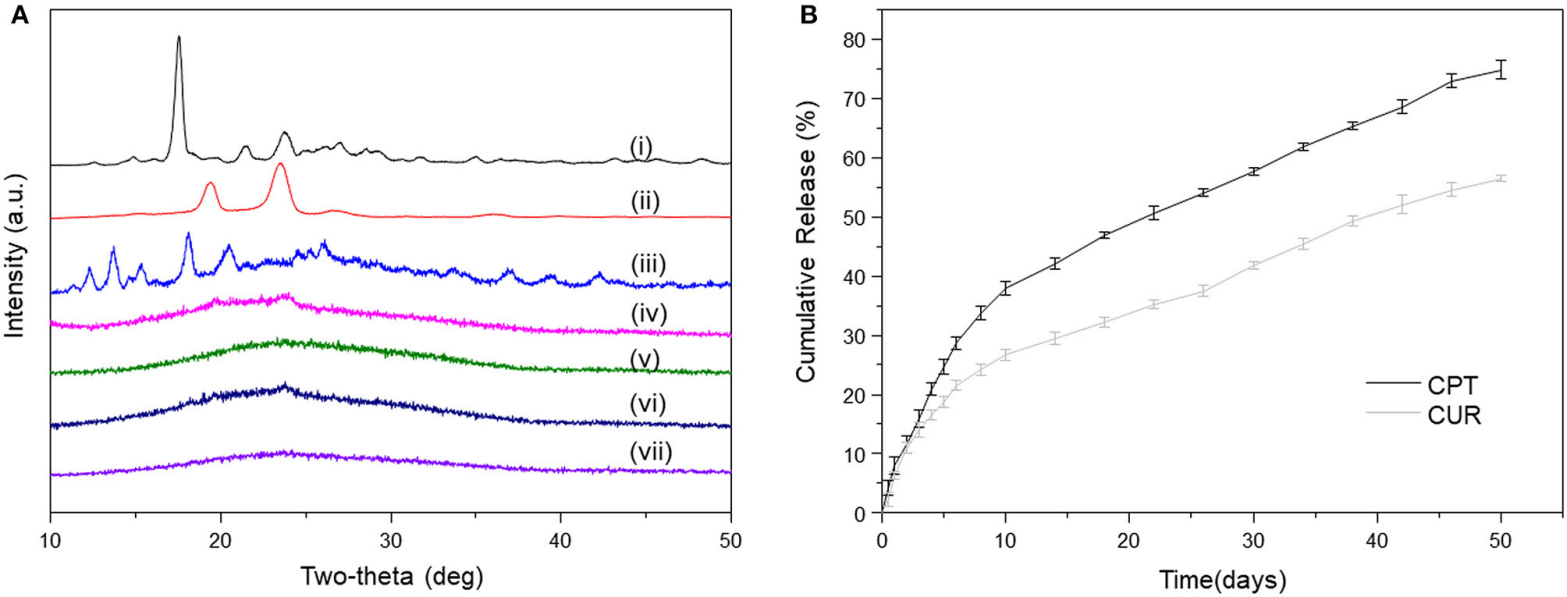
**(A)** XRD patterns of (i) pristine CUR, (ii) pristine PF127, (iii) pristine CPT, (iv) blank PF127-mesh, (v) PF127-CUR-mesh, (vi) PF127-CPT-mesh, and (vii) PF127-CPT/CUR-mesh (2:1). **(B)** *In vitro* cumulative release profiles of CPT and CUR from PF127-CPT/CUR-mesh (2:1) (*n* = 3).

As reported, crystalline states of drug molecules in DDSs feature prominently concerning the release profiles of drugs, and their amorphous state is beneficial for the constant drug release (Kim et al., 2004). Thus, we determined the crystalline states of CPT and CUR in all the meshes. As shown in Figure 3A, the XRD diffractograms of prinstine CPT, prinstine CUR, and prinstine PF127 exhibited obvious sharp and characteristic peaks between 10 and 30°, while blank PF127-mesh, PF127-CUR-mesh, PF127-CPT-mesh, and PF127-CPT/CUR-mesh (2:1) presented smooth curves. The absence of peaks for the drugs (CPT and CUR) implies that these drugs mainly exsit in a non-crystalline state within fibrous meshes. This phenomenon may be attributed to the molecular interactions among PLGA, the hydrophobic segments of PF127, and drug molecules, preventing the formation of drug crystallines.

Constant drug release from fibrous meshes is critical for localized chemotherapy. The release profiles of CPT and CUR from fibrous meshes as a function of time are presented in Figure 3B. It was found that the PF127-CPT/CUR-meshes (2:1) achieved sustained drug release within 50 days. We further found that CPT and CUR were released simultaneously from fibrous meshes with a slight initial rapid release followed by a relatively slower release phase. Moreover, a slower release rate of CUR was detected as compared with that of CPT, which was ascribed to the differences in their solubilities.

### Thermal Analyses of Meshes

**Figure 4:**
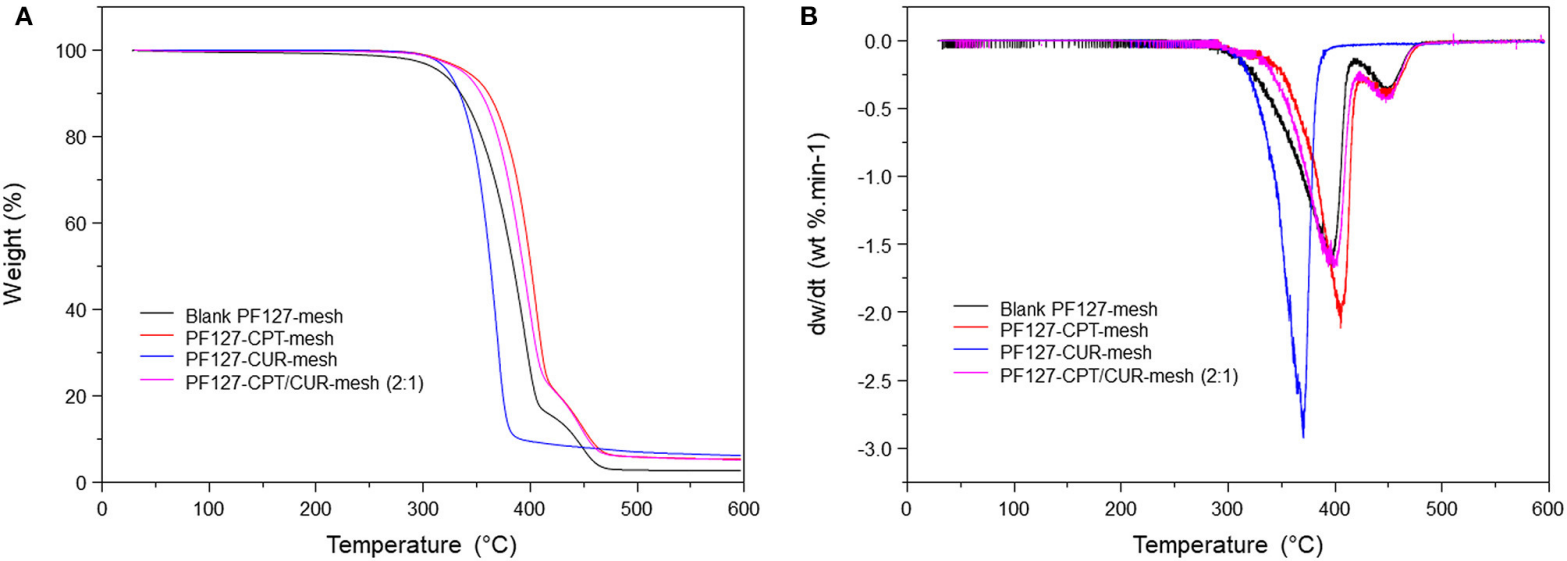
**(A)** TG curves and **(B)** DTG curves of various meshes.

TGA was used to examine the thermal properties of blank PF127-mesh, PF127-CPT-mesh, PF127-CUR-mesh, and PF127-CPT/CUR-mesh (2:1), as shown in Figure 4A. The thermogram of PF127-CUR-mesh showed an obvious stage between 320 and 380 °C with 90.9% of weight loss for strong decomposition. By comparison, thermogravimetric curves of blank PF127-mesh, PF127-CPT-mesh, and PF127-CPT/CUR-mesh (2:1) exhibited two weightlessness intervals. The first stage occurred between 320 and 410°C, which was due to the degradation of PLGA. In the first stage of thermal degradation, there were no significant changes in the contents of total weight loss for these three kinds of meshes. The second stage was concerned with the degradation of PF127 component, ranging from 410 to 430°C. The differential thermal gravity (DTG) and characteristic parameters of different fibrous meshes are presented in Figure 4B and Table 2 for the quantitative analysis of thermogravimetric curves. *T*_max_ represents the maximum degradation rate of meshes. The peaks at lower or higher temperatures corresponding to the maximum degradation rate of different samples were marked with *T*_max1_ and *T*_max2_. The *T*_max1_ of PF127-CUR-mesh was 384.7°C, which was different from the other meshes (from 396 to 406°C).

**Table 2:** Parameters of thermal degradation of different fibrous meshes.

**Fibrous mesh**	**First stage**			**Second stage**		
	**Range (**°**C)**	**T_**max1**_**	**Weight loss (%)**	**Range (**°**C)**	**T_**max2**_**	**Weight loss (%)**
Blank PF127-mesh	320–410	396.5	84.2	410–470	450.1	13.3
PF127-CPT-mesh	320–410	405.5	76.6	410–470	449.7	17.3
PF127-CUR-mesh	320–380	384.7	90.9	–	–	–
PF127-CPT/CUR-mesh (2:1)	320–410	396.8	75.3	410–470	449.1	15.3

### Anti-colon Cancer Activities of Meshes

**Figure 5:**
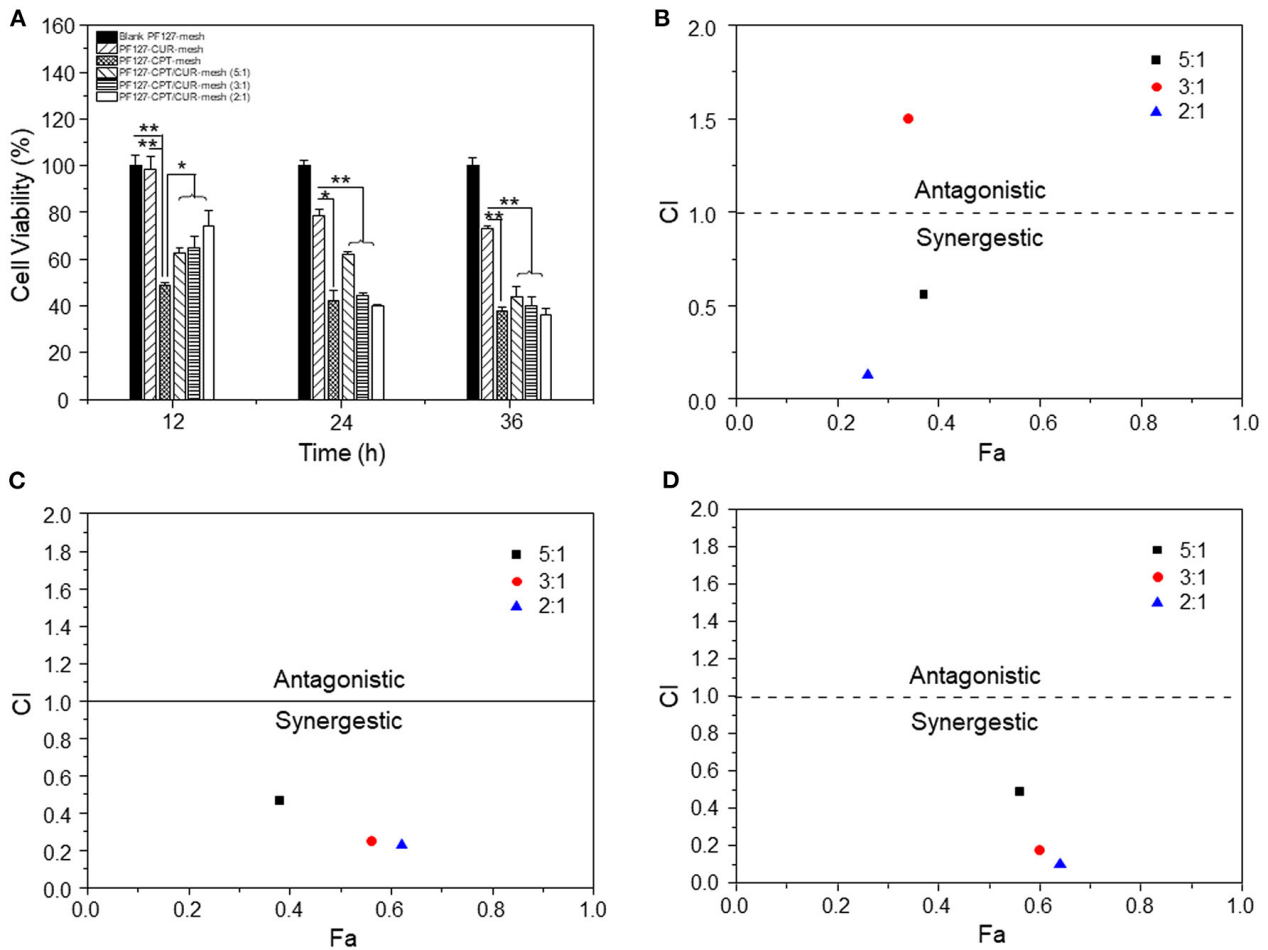
**(A)** *In vitro* anti-cancer activities of various meshes against CT-26 cells (*n* = 5; *p < 0.05 and **p < 0.01). CI values for CT-26 cells incubated with various PF127-CPT/CUR-meshes for **(B)** 12, **(C)** 24, and **(D)** 36 h, respectively.

To evaluate the anti-colon cancer activities, we treated CT-26 cells with different meshes for 12, 24, and 36 h, respectively. It was found in Figure 5A that PF127-CUR-mesh showed no significant cytotoxicity against CT-26 cells after 12 h of incubation, and it exhibited slightly anti-colon cancer activities after co-incubation for 24 and 36 h, respectively. Nevertheless, the PF127-CPT-mesh exhibited apparent toxicity on colon cancer cells since all the values of cell viabilities were <50% after incubation for 12, 24, and 36 h, respectively.

Subsequently, the synergistic effects of CPT and CUR in fibrous meshes were evaluated. We found that these effects depended on the drug ratios and time intervals (Figures 5B–D). Twelve hours after incubation, the CI values of PF 127-CPT/CUR-meshes with weight ratios of 5:1 and 2:1 were lower than 0.6, suggesting a good synergy effect for CT-26 cells treatment. After incubation for 24 and 36 h, all the CI values were <0.5, indicating that the synergistic effects of CPT and CUR were enhanced with the prolongation of incubation time. Moreover, the PF127-CPT/CUR-mesh (2:1) had the lowest CI value, indicating the best synergism effect. Mathematical models have been considered as important tools to discover the drug release kinetics of drug delivery systems (Baishya, 2017). The CPT release profile was found to be best fitted by weibull moedl and higuchi model (Supplementary Table 1). This finding suggests that the release of CPT molecules from fibrous mesh was based on CPT diffusion and matrix erosion. We also found that the CUR release profile was perfectly fitted by higuchi model (Supplementary Table 2), indicating that the release of CUR was mainly dependent on its diffusion from the mesh matrix.

## Conclusion

In this study, we employed electrospun fibers to co-deliver CPT and CUR for localized combination chemotherapy against colon cancer. The loaded drugs (CPT and CUR) were encapsulated within the fibers of the fibrous meshes, and they were released from these meshes simultaneously and sustainably. The PF127-CPT/CUR-meshes showed drug ratio-dependent synergistic chemotherapeutic effects against colon cancer cells, with meshes at a CPT/CUR weight ratio of 2:1 showing the lowest combination index value. These results obviously demonstrate that the PF127-CPT/CUR-mesh (2:1) has the potential to be processed into tubes and inserted into the colon lumen with tumor tissues for effective localized combination chemotherapy against colon cancer.

## Data Availability Statement

The original contributions presented in the study are included in the article/Supplementary Material, further inquiries can be directed to the corresponding authors.

## Ethics Statement

The animal study was reviewed and approved by Institutional Animal Care and Use Committee, Southwest University.

## Author Contributions

BX designed experiments, supervised studies, and wrote the manuscript. DX, PM, XD, and XY performed the experiments. LD, BX, and SY edited and revised the manuscript. All authors have given approval to the final version of the manuscript.

## Conflict of Interest

The authors declare that the research was conducted in the absence of any commercial or financial relationships that could be construed as a potential conflict of interest.

## References

[B1] Arnold M. Sierra M. S. Laversanne M. Soerjomataram I. Jemal A. Bray F. (2017). Global patterns and trends in colorectal cancer incidence and mortality. Gut 66, 683–691. 10.1136/gutjnl-2015-310912 26818619

[B2] Arredondo J. Baixauli J. Pastor C. Chopitea A. Hernández-Lizoain J. L. (2017). Mid-term oncologic outcome of a novel approach for locally advanced colon cancer with neoadjuvant chemotherapy and surgery. Clin. Transl. Oncol. 19, 379–385. 10.1007/s12094-016-1539-4 27496023

[B3] Baishya H. (2017). Application of mathematical models in drug release kinetics of carbidopa and levodopa ER tablets. J. Dev. Drugs 6:2. 10.4172/2329-6631.1000171

[B4] Baliga M. S. Joseph N. Venkataranganna M. V. Saxena A. Ponemone V. Fayad R. (2012). Curcumin, an active component of turmeric in the prevention and treatment of ulcerative colitis: preclinical and clinical observations. Food Funct. 3, 1109–1117. 10.1039/c2fo30097d 22833299

[B4a] Darpa P. Beardmore C. Liu L. (1990). Involvement of Nucleic Acid Synthesis in Cell Killing Mechanisms of Topoisomerase Poisons. Cancer Res. 50, 6919–6924. 1698546

[B5] Dylla S. J. Beviglia L. Park I.-K. Chartier C. Raval J. Ngan L.. (2008). Colorectal cancer stem cells are enriched in xenogeneic tumors following chemotherapy. PloS ONE 3:e2428. 10.1371/annotation/2aa6a20a-e63c-49b6-aeea-aae62435617f 18560594 PMC2413402

[B6] Ha W. Yu J. Song X. Zhang Z. Liu Y. Shi Y. (2013). Prodrugs forming multifunctional supramolecular hydrogels for dual cancer drug delivery. J. Mater. Chem. B. 1, 5532–5538. 10.1039/c3tb20956c 32261176

[B7] Han D. Sasaki M. Yoshino H. Kofuji S. Sasaki A. T. Steckl A. J. (2017). *In-vitro* evaluation of MPA-loaded electrospun coaxial fiber membranes for local treatment of glioblastoma tumor cells. J. Drug Deliv. Sci. Technol. 40, 45–50. 10.1016/j.jddst.2017.05.017

[B8] Hu J. Wei J. Liu W. Chen Y. (2013). Preparation and characterization of electrospun PLGA/gelatin nanofibers as a drug delivery system by emulsion electrospinning. J. Biomat. Sci. Polym. E. 24, 972–985. 10.1080/09205063.2012.728193 23647252

[B9] Kim K. Luu Y. K. Chang C. Fang D. Hsiao B. S. Chu B.. (2004). Incorporation and controlled release of a hydrophilic antibiotic using poly(lactide-co-glycolide)-based electrospun nanofibrous scaffolds. J. Control Release 98, 47–56. 10.1016/j.jconrel.2004.04.009 15245888

[B10] Kim K. Yu M. Zong X. Chiu J. Fang D. Seo Y. S.. (2003). Control of degradation rate and hydrophilicity in electrospun non-woven poly(d,l-lactide) nanofiber scaffolds for biomedical applications. Biomaterials 24, 4977–4985. 10.1016/S0142-9612(03)00407-1 14559011

[B11] Lashof-Sullivan M. McSweeney A. L. Ting D. T. Kim M. P. Tzeng C. W. D. Indolfi L. (2019). Targeted and sustained drug delivery therapy for localized pancreatic cancer: *in vivo* validation in porcine models. Cancer Res. 79:B26. 10.1158/1538-7445.PANCA19-B26

[B12] Liu J. Jiang Z. Zhang S. Saltzman W. M. (2009). Poly(omega-pentadecalactone-co-butylene-co-succinate) nanoparticles as biodegradable carriers for camptothecin delivery. Biomaterials 30, 5707–5719. 10.1016/j.biomaterials.2009.06.061 19632718 PMC2774808

[B13] Ma P. Gou S. Ma Y. Chen Q. Chen J. Xiao B.. (2019). Modulation of drug release by decoration with pluronic F127 to improve anti-colon cancer activity of electrospun fibrous meshes. Mat. Sci. Eng. C Mater. 99, 591–598. 10.1016/j.msec.2019.01.130 30889734

[B14] Ma P. Gou S. Wang M. Chen J. Hu W. Xiao B. (2018). Knitted silk fibroin-reinforced bead-on-string electrospun fibers for sustained drug delivery against colon cancer. Macromol. Mater. Eng. 303:1700666. 10.1002/mame.201700666

[B15] Martino E. Della Volpe S. Terribile E. Benetti E. Sakaj M. Centamore A.. (2017). The long story of camptothecin: From traditional medicine to drugs. Bioorg. Med. Chem. Lett. 27, 701–707. 10.1016/j.bmcl.2016.12.085 28073672

[B16] Mirjalili M. Zohoori S. (2016). Review for application of electrospinning and electrospun nanofibers technology in textile industry. J. Nanostructure Chem. 6, 207–213. 10.1007/s40097-016-0189-y

[B17] Nelson K. M. Dahlin J. L. Bisson J. Graham J. Pauli G. F. Walters M. A. (2017). The Essential medicinal chemistry of curcumin. J. Med. Chem. 60, 1620–1637. 10.1021/acs.jmedchem.6b00975 28074653 PMC5346970

[B18] Ramasamy T. Kim J. H. Choi J. Y. Tran T. H. Choi H. G. Yong C. S.. (2014). pH sensitive polyelectrolyte complex micelles for highly effective combination chemotherapy. J. Mater. Chem. B. 2, 6324–6333. 10.1039/C4TB00867G 32262149

[B19] The Colon Cancer Laparoscopic or Open Resection Study Group (2009). Survival after laparoscopic surgery versus open surgery for colon cancer: long-term outcome of a randomised clinical trial. Lancet Oncol. 10, 44–52. 10.1016/S1470-2045(08)70310-3 19071061

[B20] Thompson C. J. Chase G. G. Yarin A. L. Reneker D. H. (2007). Effects of parameters on nanofiber diameter determined from electrospinning model. Polymer 48, 6913–6922. 10.1016/j.polymer.2007.09.017

[B21] Wiranowska M. Toomey R. Falahat R. Alcantar N. (2019). Design for a flexible localized drug delivery system. Cancer Res. 79:3615. 10.1158/1538-7445.SABCS18-3615

[B22] Xiao B. Han M. K. Viennois E. Wang L. Zhang M. Si X.. (2015a). Hyaluronic acid-functionalized polymeric nanoparticles for colon cancer-targeted combination chemotherapy. Nanoscale 7, 17745–17755. 10.1039/C5NR04831A 26455329 PMC4618760

[B23] Xiao B. Si X. Han M. K. Viennois E. Zhang M. Merlin D. (2015b). Co-delivery of camptothecin and curcumin by cationic polymeric nanoparticles for synergistic colon cancer combination chemotherapy. J. Mater. Chem. B. 3, 7724–7733. 10.1039/C5TB01245G 26617985 PMC4662402

[B24] Xiao B. Zhang M. Viennois E. Zhang Y. Wei N. Baker M. T.. (2015c). Inhibition of MDR1 gene expression and enhancing cellular uptake for effective colon cancer treatment using dual-surface-functionalized nanoparticles. Gastroenterology 148, S638–S638. 10.1016/S0016-5085(15)32149-1 25701040 PMC4339818

[B25] Xie C. Li X. Luo X. Yang Y. Cui W. Zou J.. (2010). Release modulation and cytotoxicity of hydroxycamptothecin-loaded electrospun fibers with 2-hydroxypropyl-beta-cyclodextrin inoculations. Int. J. Pharm. 391, 55–64. 10.1016/j.ijpharm.2010.02.016 20170717

