# Supplementary material for: Pluronic F127-Modified Electrospun Fibrous Meshes for Synergistic Combination Chemotherapy of Colon Cancer
Source: Front Bioeng Biotechnol. 2021 Feb 16;8:618516. doi: 10.3389/fbioe.2020.618516 (PMC7921460; doi:10.3389/fbioe.2020.618516)
Supplement: Supplementary file 1 [file Data_Sheet_1.docx]

**Supporting Information**

**Pluronic F127-modified electrospun fibrous meshes for synergistic combination chemotherapy of colon cancer**

Dengchao Xie^1,2†^, Panpan Ma^3†^, Xin Ding^1,4^, Xiao Yang^1^, Lian Duan^1*^, Bo Xiao^1,4*^ and Shixiong Yi^1*^

^1^ State Key Laboratory of Silkworm Genome Biology, College of Sericulture, Textile and Biomass Sciences, Southwest University, Beibei, Chongqing, China

^2^ College of Food Science, Southwest University, Beibei, Chongqing, China

^3^ Chemical and Biological Technologies for Health Unit, School of Pharmacy, CNRS UMR8258, INSERM U1267, Université de Paris, Paris, France

^4^ Ministry of Agriculture and Rural Affairs Key Laboratory of Sericultural Biology and Genetic Breeding, College of Sericulture, Textile and Biomass Sciences, Southwest University, Beibei, Chongqing, China

^†^These authors have contributed equally to this work.

^*^Correspondence: Lian Duan (duan19850420@163.com), Bo Xiao ([bxiao@swu.edu.cn](mailto:bxiao@swu.edu.cn))，and Shixiong Yi (yishixiong@swu.edu.cn)


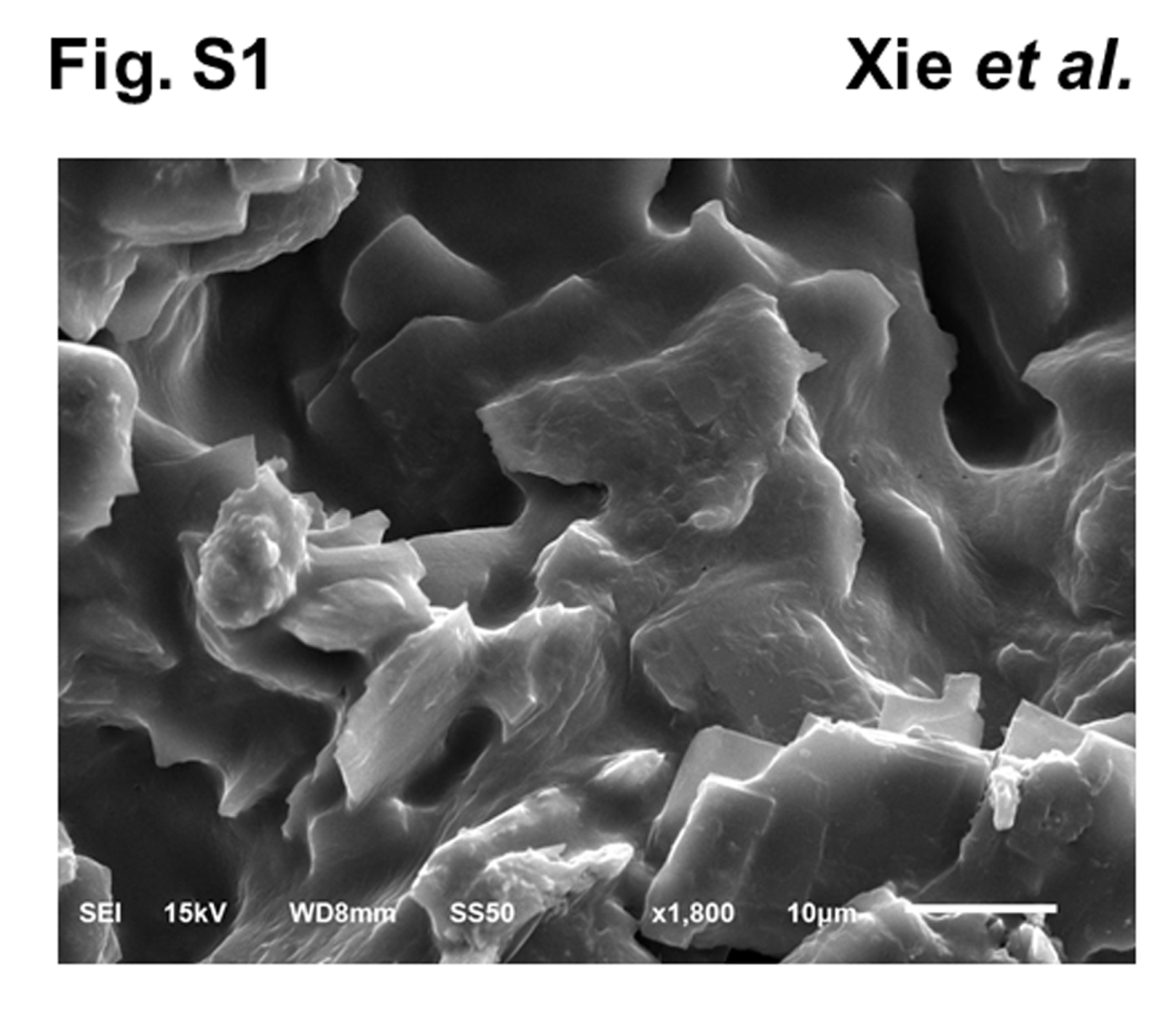


**Figure S1.** SEM image of PF127-CPT/CUR-mesh (2:1) after incubation in simulated colonic fluid (pH 6.2) for 49 days.

**Table S1.** Regression equations of the release profiles of CPT from fibrous meshes.

| Model | Regression equation | r^2^ |
| --- | --- | --- |
| Zero-order kinetics | Q= 0.1623 + 0.0131t | 0.9252 |
| First-order kinetics | Ln(1-Q)= -0.1329 - 0.0252t | 0.9846 |
| Weibull | LnLn[1/(1-Q)]= -2.5108 + 0.7186lnt | 0.9932 |
| Niebergull | (1-Q)^1/2^= 0.9229 - 0.009t | 0.9625 |
| Hixcon-crowell | (1-Q)^1/3^= 0.9503 - 0.0067t | 0.9717 |
| Peppas | LnQ= -2.4922 + 0.5893lnt | 0.9783 |
| Higuchi | Q=d+ct^1/2^= -0.0083 + 0.109t^1/2^ | 0.9913 |

**Table S2.** Regression equations of the release profiles of CUR from fibrous meshes.

| Model | Regression equation | r^2^ |
| --- | --- | --- |
| Zero-order kinetics | Q= 0.119 + 0.0097t | 0.9460 |
| First-order kinetics | Ln(1-Q)= -0.1087 - 0.0149t | 0.9794 |
| Weibull | LnLn[1/(1-Q)]= -2.7997 + 0.6715lnt | 0.9674 |
| Niebergull | (1-Q)^1/2^= 0.9422 - 0.006t | 0.9659 |
| Hixcon-crowell | (1-Q)^1/3^= 0.9621-0.0043t | 0.9711 |
| Peppas | LnQ= -2.7992+0.5912lnt | 0.9496 |
| Higuchi | Q=d+ct^1/2^= -0.0037+0.0797t^1/2^ | 0.9913 |
